# Perspective of Family Members of Transitions to Alternative Levels of Care in Anglo-Saxon Countries

**DOI:** 10.1155/2018/4892438

**Published:** 2018-02-21

**Authors:** C. Merla, A. Wickson-Griffiths, S. Kaasalainen, V. Dal Bello-Haas, L. Banfield, T. Hadjistavropoulos, E. Di Sante

**Affiliations:** ^1^School of Nursing, McMaster University, Hamilton, ON, Canada; ^2^Faculty of Nursing, University of Regina, 3737 Wascana Parkway, Regina, SK, Canada S4S 0A2; ^3^School of Nursing, McMaster University, 1280 Main Street West, HSC 3N25F, Hamilton, ON, Canada L8S 4K1; ^4^School of Rehabilitation Science, McMaster University, IAHS 403/E, 1400 Main Street West, Hamilton, ON, Canada L8S 1C7; ^5^Faculty of Health Sciences, McMaster University, 1280 Main Street West, Hamilton, ON, Canada L8S 4K1; ^6^Department of Psychology and Centre on Aging and Health, University of Regina, 3737 Wascana Parkway, Regina, SK, Canada S4S 0A2; ^7^School of Nursing, McMaster University, 1280 Main Street West, HSC 3H28, Hamilton, ON, Canada L8S 4K1

## Abstract

This scoping review explores circumstances surrounding the decision about, and eventual experience of, transitioning older adults into alternative levels of housing (ALH), such as long-term care. This topic is examined from a family member perspective, given their exposure and involvement in the care of older adult relatives during this transitional period. The scoping review methodology is based on the framework of Arksey and O'Malley and subsequent recommendations from Levac, Colquhoun, and O'Brien. Approximately 470 articles were reviewed covering the period between 2000 and November 2014; 37 articles met inclusion criteria. A temporal organization of themes was used to describe the experiences of family members in the pretransition, active transition, and posttransition periods of moving older adult relatives into ALH. This paper highlights the transitional period as a time of crisis, with a lack of planning, support, and transparent discussion. This study identifies a need for future research on the potential benefits of family support groups, interim transitional housing options, different models of ALH, changing roles in the posttransition period, and the need for a comprehensive list of housing options for older adults. Results have the potential to inform policy/practice and improve the lives of older adults and their family.

## 1. Introduction

By 2050, individuals aged 60 years and older will comprise approximately 22% of the world's population [1]. As the number of older adults increases dramatically, countries will see a greater percentage of older adults living in independent dwellings in need of support [1]. Statistics Canada [2] reported that 92.1% of nearly 5 million adults aged 65 years and older lived in private dwellings, and 7.9% lived in collective dwellings, such as long-term care homes. Data has highlighted that while older adults may initially live independently at home, advanced age coincides with greater needs and increased likelihood of moving into a collective dwelling that provides ongoing assistance and support and professional health monitoring and treatment [2].

Until a final decision is made to transition into a collective dwelling, support and assistance from others play a vital role in maintaining dignity and preserving a meaningful life [2]. The Canadian Caregiver Coalition [3] describes informal caregiving as a universal phenomenon that includes the provision of ongoing care, in the absence of payment, for family members and friends who require additional support due to diminishing physical ability, debilitating cognitive health, and/or a chronic life-limiting ailment [3]. Most often it is family members who initially assume the role of informal caregiver.

It is well known that informal caregiving responsibilities can place a significant demand on family members, especially members of the “sandwich generation” who must juggle care demands of child-rearing, employment, personal interests, and care for their aging friends and relatives. Given their exposure and involvement in the care of older adults' relatives, family members possess a unique perspective that has the potential to enrich our understanding of the circumstances surrounding the decision about, and eventual experience of, transitioning older adults into alternative levels of housing, such as long-term care. Greater recognition of the overall experience and the issues involved in transitioning older adults into alternative levels of housing from a family member perspective is thus an important area of research. This research area has the potential of informing policy and practice and ultimately improving the lives of older adults and their family members.

Over the past two decades, there has been a modest increase in the number of research studies exploring the experiences of family caregivers during the transition process of a loved one into alternative levels of housing support. Three literature reviews have been conducted on this topic [4–6]. All three literature reviews were published more than a decade ago [4, 6, 7] and therefore lack a contemporary representation of current literature in this area of research. The paucity of research in this area necessitates a comprehensive review of the more recent literature in order to understand the current state of knowledge, to identify key experiences, and to develop recommendations for future research. The purpose of this scoping review is to examine the experiences of family members as they help to transition older adults they have been caring for from one level of housing (e.g., home or hospital) into an alternate level of housing with more support (e.g., nursing home or long-term care home).

## 2. Methods

A scoping review differs from systematic reviews, in that the latter are more likely to identify and address more well-defined and focused questions as well as judge the quality of included studies and are limited to specific designs. A scoping review is the preferred type of review to focus on key experiences of family members as they help to transition their older adult relatives into alternative levels of housing (e.g., long-term care, nursing home, retirement homes, and assisted living facilities). In keeping with the tenants of scoping reviews, our presentation does not include evaluation of the methodological quality of included articles.

The methodology of this scoping review is based on the framework of Arksey and O'Malley [8] and subsequent recommendations from Levac et al. [9]. Arksey and O'Malley [8] recommend six stages in their methodological framework for conducting scoping reviews in order to ensure a comprehensive and objective approach to the identification of research gaps in existing literature and exploration of the state of knowledge. The stages include (1) identifying the research question; (2) identifying relevant studies; (3) study selection; (4) charting the data; (5) collating, summarizing and reporting the results; and (6) consultation.

Arksey and O'Malley [8] suggest starting with a wide approach or broad research question to generate breadth and coverage. Once a general sense of the volume and scope of the field has been gained, then tighter parameters around the research questions can be set if needed. This scoping review addressed the following research question: “What are family caregiver's experiences when transitioning older adults into alternate levels of housing in Anglo-Saxon countries?”

According to Arksey and O'Malley [8] the goal of this stage is to be as comprehensive as possible in identifying primary studies. Thus, in consultation with a librarian from McMaster University (LB), key electronic databases and search terms were identified in September 2014. Databases included the Cumulative Index to Nursing and Allied Health Literature, Medline, Ovid, and Ageline. Search terms were identified for four categories including place of residence (e.g., nursing home, community dwelling, and assisted living), older adult (e.g., elderly person, nursing home resident), transition (e.g., transfer, placement), and decision-making. Search terms within each category were combined with the “OR” command, and the subsequent results were merged with the “AND” command. The search was limited to literature published in English between 2000 and November 2014. Our sample was restricted to only articles published in English as this was the only shared working language of all of the authors. We chose to only include articles in English from Anglo-Saxon countries because cultural influences may have had differing impacts on results coming from non-Anglo-Saxon countries. It is beyond the scope of this paper to analyze all of the articles with respect to their cultural influence. Future research should explore this at a more in-depth level. Hand searching of key journals and citation tracking of included articles was conducted to identify additional literature not captured in the initial search.

Selection of studies should be an iterative process of searching the literature, refining the search strategy, and reviewing articles for study inclusion. The following process was used to select studies. First, the database search returned 433 unique results, after accounting for 309 duplicates. Second, examination of key articles produced an additional 37 results. All results were included in the title and abstract review. Results were included for full-text review if they focused on a family members' experience or decision to transition their older adult relative to an alternate type of permanent housing. Older adult relatives were considered to be 65 years and older. The transition had to be to housing that would provide more assistance or a higher level of care for the older adult than their current dwelling (e.g., transitioning from an assisted living facility to a nursing home). To be included in our review, results had to be published as an article rather than other types of publications such as a thesis or abstract.

A single reviewer (AWG) completed the initial title and abstract review, rating each search result as relevant or not. A second reviewer then reviewed results where relevance was not clear (SK). In total, 37 articles were identified for full-text review.

Two reviewers (MT, CM) extracted key items or variables from the studies reviewed in order to address the research question. An initial data extraction table was created by MT and included the following headings: general information (e.g., first author of study, year of publication, publication type, and country of origin); study design (e.g., qualitative, quantitative, mixed methods, and reviews); study aim; and participants (e.g., study participants, sample size, sex, age, persons with dementia included, and diagnosis). Data extraction was conducted in consultation with SK and AWG.

To analyze the extracted data, a combination of tabular summaries, content analyses, and team discussion were used. CM created a summary table of key themes generated from the literature and categorized under the following headings: themes, subthemes, and quotes/notes. The literature was further organized into time periods, including the pretransition, active transition, and posttransition experiences of family members; and information was collated into key themes that traversed the literature. This summary was created in consultation with SK and AWG.

## 3. Results

### 3.1. Article Characteristics

**Figure 1:**
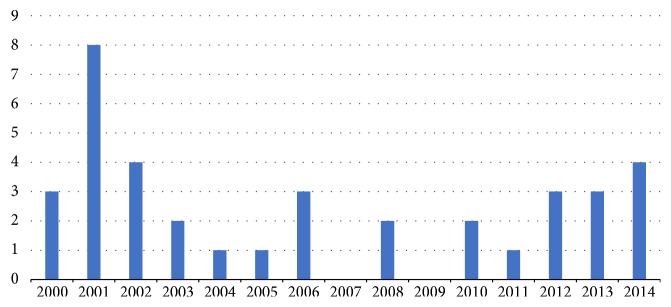
Year of article publication.

**Figure 2:**
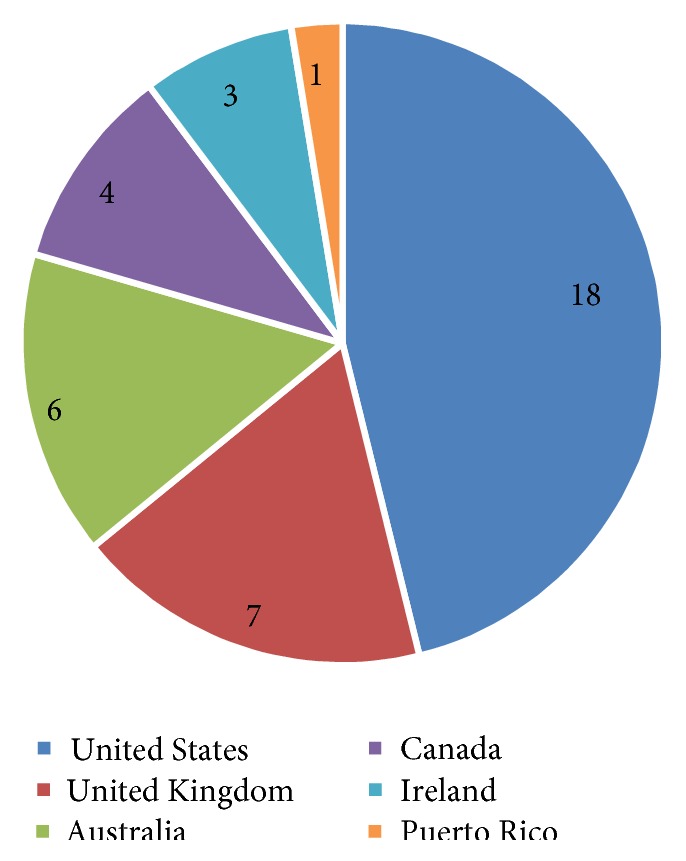
Study country of origin. *Note*. Two articles were based on research conducted in two countries equaling 39 countries.

A total of 37 articles were identified as relevant and met the inclusion criteria. Of the 37 articles, two were editorials [10, 11], three were literature reviews [4–6], and the remaining 32 articles consisted of qualitative (*n* = 23), quantitative (*n* = 5), and mixed method study designs (*n* = 2) and secondary analyses (*n* = 2). Articles were published from 2000 to 2014, as demonstrated in Figure 1, which highlights the distribution of articles published per year.  Figure 2 outlines the country of each publication. The majority of included articles were published in the United States (*n* = 18).

A variety of settings were involved in transitions to alternative levels of housing. Given the number of countries represented in this scoping review, a variety of similar descriptors were used. These descriptors included transitions from the older adult's independent dwelling, a family members home, hospital, long-term care, assisted living, congregate housing, retirement home, and care facility, to assisted living home, nursing homes, nursing facilities, long-term care facilities, aged care facilities, care homes, and institutional homes. In all cases, individuals were transitioning into new housing with additional support.

### 3.2. Key Findings

A temporal organization of themes [12–14] was used to describe the experiences of family members in the pretransition, active transition, and posttransition periods of moving their older adult relatives into alternative levels of housing (ALH). It is important to note that while a temporal perspective was used, phases of the transition process have been well documented in the literature as “haphazard,” “lengthy,” and “troubling” for many family members and older adults alike; therefore, in many instances this process was nonlinear, complex, and potentially traumatic for all involved [4, 7, 14–17]. According to Aubrey-Fletcher [11] the transition process can be eased through careful guidance, planning, and support. Unfortunately, careful planning, guidance, and support are not a reality for many individuals and their family members.

The pretransition period includes information on planning processes and outcomes, precipitating factors that lead to transition, emotions expressed by family members prior to the decision to transition, and primary reasons against the transition. The active transition section of this review explores feelings of being pressured, lacking choice and being rushed, informal and formal influences, and the quality of nursing homes as key themes highlighted in the literature. Furthermore, the posttransition period is presented as a period of emotional and role adjustment and incorporates key factors contributing to familial satisfaction and adjustment.

### 3.3. Pretransition Period

The pretransition period encompasses the time prior to the decision to transition an older adult into an ALH. Initially, family members may or may not be aware of the need to transition their older adult relative; however, gradually or through crisis, they come to realize the need for additional support in order to maintain wellbeing and preserve quality of life. For this reason, this phase is described as the pretransition time period initiating the decision against or for the transition.

### 3.4. Planning during Pretransition Period

The pretransition process is described in the literature as a time characterized by two extremes: limited planning and crisis transitioning or careful planning and gradual transitioning to ALH. Each of these extremes is discussed next.

### 3.5. No Planning (Crisis)

The decision to transition older adults into ALH is most often initiated by a crisis situation, such as an acute admission to hospital or the sudden deterioration of an older adult's and/or caregiver's health [4, 7, 10, 16, 18–24]. Castle [12] found, using a sample size of 306 older adult residents, that 78% of nursing home placements occurred following hospitalization. Cheek et al. [24], Magilvy and Congdon [25], and Caldwell et al. [18] reported similar findings indicating that very few proactive nursing home choices are being made prior to crisis situations.

Several reasons for a lack of planning for ALH were found. Many family members perceive ALH as a last resort and hold off on admission for as long as possible until they can no longer manage, alternative resources and services fail, and/or there are no other options [2, 7, 10, 18, 22]. One participant from Caldwell et al.'s [18] study of 27 caregivers of older adults with dementia eloquently stated that “the last resort is nursing home… nobody likes to be admitted to nursing home. We hang on until we have no choice” (p. 418).

In some cases, family members may not even discuss or see the need for entry into ALH until a crisis occurs and catches their attention [4, 18, 20]. According to one nurse from Magilvy and Congdon's [25] study of 175 older adults, healthcare providers and community leaders, “it usually takes a crisis or an admission to a hospital to get families thinking and planning; usually no planning is done until it is crisis time, and then families can't always involve the person in decision-making. People can't make choices for themselves if they are acutely ill or in crisis. And at that point, family members don't always agree.” This quote not only highlights the crisis situation but also points towards some of the associated issues with transitions that will be discussed. Importantly, a lack of planning can result in time pressures and force quick decisions that may result in subsequent feelings of regret and issues with posttransition adjustment [4, 26].

### 3.6. Planning and Gradual Transitions

Planning for the transition of older adults into ALH is generally regarded as a more positive experience and is described as an “anticipatory” or gradual transition [4, 12, 18]. Proactive planning affords family members and older adults sufficient time to visit facilities and make informed decisions on future living arrangements [12, 18]. According to Castle [12] family member satisfaction improves significantly when family members visited facilities, long-term care was considered an acceptable option for housing, and a significant amount of time was spent on choosing a facility. In fact, those who spent more than average time looking for a facility (more than 133 days) were associated with 118% odds that they would be very satisfied with the facility compared with those who spent less than average time [12]. Individuals who engage in proactive planning often became gradually aware of their inability to continue providing care and/or recognize that the health of the older adult will continue to deteriorate and result in greater care needs [18, 27].

### 3.7. Precipitating Factors Leading to the Transition

Several factors that precipitate the transition of older adults into ALH were found. Penrod and Dellasega [21] describe these precipitating factors as initiating an evaluation of demands and resources among caregivers, resulting in the decision to transition older adults due to inadequate caregiving situations. Health and deterioration of the older adult and/or family caregiver, as well as influences from others, were identified in the literature as primary factors precipitating the transition [6, 7, 10, 12, 16, 18, 19, 22, 25, 27–30].

### 3.8. Health and Deterioration of Older Adults

Health deterioration precedes the process of relocating older adults into ALH [6, 7, 12, 18, 19, 27, 31]. Castle's [12] study of 306 older adults revealed that resident health factors were the most important reasons for nursing home placement in 60% of cases. Difficulty managing the care needs and safety concerns of older adults with dementia were associated with an increased likelihood of transitioning into ALH [6, 18, 28, 29, 32, 33]. Uncontrollable, dangerous dementia-related behaviours included wandering and becoming lost, hallucinating, paranoia, sexual assault, forgetting medication, inadequate nutrition, and accidents in the home, such as fires or falls [18, 27]. Bond and Clark [28] studied 163 spousal caregivers and found that dementia severity was a strong indicator of the need to transition older adults into formalized care. As dementia symptoms progressed, caregivers were more likely to place loved ones in ALH to address advanced care needs [28].

Physical health concerns, such as hypertension, diabetes, fractures, stroke, incontinence, vision loss, falls, and immobility further necessitated transition decisions [4, 6, 16, 22, 27]. In Nolan and Dellasega's [29] study of 102 care providers, seven out of 10 nursing home admissions were influenced by the concern that older adults were unable to perform self-care and caregivers' inability to address increasing physical needs. Multiple mental, physical, and behavioural comorbidities exasperated care needs and were strongly linked to institutionalization rates [18, 27, 28]. Furthermore, uncertainty of prognosis and rehabilitative capacity of older adults enabled caregiver efforts to seek formal long-term care services [21].

### 3.9. Health and Deterioration of Family Caregivers

Deteriorating physical and mental health status of family caregivers also precipitated transitions to ALH [10, 16, 18, 21, 27, 28, 30]. Caregiver health concerns included a general awareness of deteriorating health, increased fatigue, pain, arthritis, fractured bones, surgery, receiving a cancer diagnosis, and experiencing a heart attack or stroke [18, 27]. Mental health concerns included depression, social isolation, anxiety, and stress among family members [10, 16, 27, 28].

According to Buhr et al. [30] when caregiver health was the primary reason for seeking formalized care, caregivers were more likely to report lower self-perceived health, more sick days at work and visits to the doctor, more medications, and comorbid conditions. In Buhr et al.'s [30] study of 2,268 informal caregivers, individuals were also more likely to have a spouse, lower income, lower life satisfaction, and higher stress symptoms, highlighting the complexity of caregiving and its intricate relationship with the social determinants of health.

### 3.10. Time-Consuming Duties and Impact on Social Life

Progressive decline in the physical and mental health status of older adults intensifies caregiver roles and responsibilities [18, 25]. Family caregivers who seek formal care for older adults report feeling stressed by the burden of caregiving, particularly when responsibilities interfere with employment, family relationships, and engagement in social activities [16, 18, 28]. Taken together these factors contribute to family caregivers' decision to transition older adults into ALH.

### 3.11. Lack of Informal and Formal Support

Family caregivers indicate that they receive little support from relatives, community members, healthcare professionals, and the government when caring for older adults [10, 16, 18, 19]. While there is literature supporting the idea that family and friends can be a source of diversion from the stress of performing caregiver duties [34], the notion that caregivers are being “exploited” or “stretched until the end” is well documented and further illustrated in Liken's [10] finding that family caregivers feel alone in performing their tasks and burdens. They feel as though friends and family members lack an appreciation of their caregiver burden. Mamier and Winslow have shown that a lack of familial assistance for spousal and child caregivers results in the use of formal care options to not only relieve caregiver burden but also provide more skilled care for older relatives [16].

### 3.12. Influence from Others

Despite the burden of caregiving, the literature suggests that many family caregivers are reluctant to independently choose nursing home placement for older relatives [20, 29, 31]. In fact, according to Ryan and Scullion [7] many family members try to distance themselves from the decision and instead act on advice from healthcare professionals, such as general practitioners, or other family members and friends [18–20, 25, 29, 31]. This advice is viewed as important when caregivers begin processing their options for care and in some cases gives caregivers “permission” to seek formal support by justifying or legitimizing acceptability of the transition and attenuating feelings of guilt [18, 21, 25, 31].

### 3.13. Emotions Expressed by Family Caregivers prior to Making the Decision

The pretransition process is an emotionally turbulent time for many family caregivers who are exposed to complex emotions ranging from ambivalence and apprehension to guilt, powerlessness, worry, and a deep sense of loss [4, 21, 23, 24, 35–37]. Feelings of ambivalence are particularly prevalent during this time period and well documented in the literature. According to Penrod and Dellasega [21] ambivalence among family caregivers stems from a conflict between the “ideal” caregiver and the harsh reality of providing care while juggling other responsibilities.

A few authors referenced ambivalence experienced by family caregivers as an individual struggle to weigh positives with negatives during the transition process [36]. In Gill and Morgan's [36] study of 12 adult daughters, all reported ambivalence regarding the decision to transition, their parent's loss of independence, changing relationships with parents, and difficult but necessary role reversals. They reported continuously weighing the positives and negatives throughout the whole process and this continued even after the transition [36]. Furthermore, Gill and Morgan [36] found that communication with parents regarding the decision to transition was a major source of ambivalence, and this led to indecision among family members, rushed decision-making, a lack of family communication, and even paternalistic behaviour among children.

Family caregivers also experienced a deep sense of loss and/or anger during the pretransition phase [25, 36]. Gill and Morgan [36] reported that physical and mental decline and a loss of independence influenced adult daughters to believe that there was no chance of retaining the highly valued parent-child relationships they had enjoyed for many years. This essentially meant that the parents they had known previously would never come back [36].

### 3.14. Reasons against Transitioning into ALH

The pretransition process has been described as a period of ambivalence and a process of weighing advantages and disadvantages of ALH transitions. This section includes literature on the key reasons why family members decide against or delay transition.

### 3.15. Family Conflict

Conflict between family caregivers and older adults or other relatives can prevent or delay transition to ALH [15, 18, 19, 31, 32, 35, 38]. Several studies report that a key deterrent in the transition process is the objection to transition among older adults [18, 19, 31].

Conflict between siblings or other family members also causes a great barrier for caregivers when deciding to place older adults in formal care [15, 18, 35, 38]. The literature on familial conflict during the pretransition phrase depicts primary caregivers as having to negotiate with family members and often delay the transition in order to appease preferences of family members who are unavailable when their support is needed [35, 38].

### 3.16. Cultural Expectations and a Sense of Duty

Cultural expectations placed on family caregivers prevent them from not only discussing the need for the transition but also initiating the process of relocation to ALH due to fear of dishonouring their family by failing to perform filial duties [4, 10, 16, 18, 26, 39]. Crist [39] uses the term “familism” to describe these cultural expectations. In essence, familism refers to the belief that family supersedes the needs of the individual and that children must help their parents in later life in order to reciprocate the sacrifices made by their parents during childhood [10, 39, 40]. These expectations create tension between perceived obligations and wanting to honour the wishes of older adults to remain at home with reaching personal limits and juggling multiple responsibilities [16, 26]. Thus, family caregivers feel pressured to continue providing care even when it results in great personal costs [4].

### 3.17. Cultural Competency of Homes

Cultural competency in ALH was identified as another reason for deferring the transition. According to Crist [39] Mexican American older adults and family members in their study viewed services as culturally insensitive, discriminatory, and lacking qualities of politeness, warmth, respect, person-oriented relationships, and empathy. Johnson and Tripp-Reimer [6] further supported this finding by noting that older adults, who are members of ethnic minority groups, and family members may fear prejudicial treatment and exclusion due to cultural differences and thus avoid transitioning into ALH.

### 3.18. Negative Perceptions of Long-Term Care

Some caregivers decide against transitioning older adults into ALH because of negative perceptions of nursing homes and the belief that they can ensure a better quality of life at home for their older relative [18, 26, 39, 41]. According to Caldwell et al. [18] some caregivers prefer to keep older adults with dementia at home because they can enjoy their life more by having, for example, the freedom to choosing outings and meals. One caregiver stated that “it's just too early stage to take her to the nursing home. Reason being that one, going to nursing home is like going to jail… so food, she can enjoy good food, she can have the general basic living, what other people do, she does some little shopping for herself, and doing all this exercise, right, it helps her brain” (18: p. 418).

### 3.19. Suitability of Facilities

Suitability of facilities and timing of transitions were perceived as another barrier preventing relocation to ALH. In terms of suitability, issues with affordability of appropriate support, inability for homes to accommodate spousal partners, and providing care for individuals with low to moderate dementia symptoms prevented transitions from occurring [16, 18, 41]. According to one caregiver from Caldwell et al.'s [18] study of 27 caregivers of people with dementia, “it's a long waiting process as well. Especially for the low care period, I think that was very hard… it's almost like oh, you know, let's let them deteriorate until they're high care and then let them in” (CR16: p. 419). This highlights some of the issues with availability of suitable facilities for people with a variety of needs and preferences.

### 3.20. Summary of Section

The pretransition period is characterized by two extremes of planning: gradual planning and crisis planning. While gradual planning is generally perceived more positively in the literature, crisis situations are the most common initiating factor and translate into difficult transitions for family members. Health deterioration of older adults and family members and influence from formal and informal networks are the key precipitating factors for transitioning into ALH. The pretransition period is also described as an emotionally turbulent time, characterized by ambivalence, guilt, powerlessness, apprehension, worry, and a deep sense of loss. Furthermore, there are several factors that can delay or deter the decision to transition, namely, familial conflict, cultural expectations regarding filial piety, negative perceptions of long-term care, and the suitability of nursing homes in meeting the needs and preferences of older adults and their family members.

### 3.21. Active Transition Period

The active transition period represents the time following the decision to transition older adults into ALH and includes the process of accessing services and information and determining the location for alternate housing. Family members' experiences, influences, and determining factors for choosing housing are discussed.

### 3.22. Feeling Pressured and Rushed

The active transition period has been described as a time filled with pressure and limited choice and control [19–21, 24, 35]. It is a time when family caregivers feel rushed by the “system” and pushed along a track set out by formal caregivers and health professionals with unequal access to information and a lack of support [4, 7, 12, 15, 18, 19, 21, 25, 26, 29, 35, 42]. Penrod and Dellasega [21] effectively liken the transition process to an obstacle course wherein family members are forced to “jump hoops” in order to safely complete the transition of older adult relatives.

### 3.23. Lacking Genuine Choice and Control

A lack of choice and control during the active transition stage is a dominant theme in the literature and traverses interactions with healthcare professionals and service providers, while also intersecting with the search and selection process for suitable homes and access to supporting information to facilitate the transition. According to Cheek and Ballantyne [35], many family members report feelings of powerlessness during the transition process, and this leads to family conflict and hostility.

A lack of communication between healthcare providers and family members can contribute to feelings of powerlessness and family conflict. It can also lead to miscommunication with older adults leaving them feeling unwanted and burdensome and troubled with the decision to return home or move to a new environment.

### 3.24. Feeling Rushed by Hospital/Service Providers/Long-Term Care Homes

Limited communication between family members and healthcare providers also impacts decision-making by placing time pressures on family members to move older adult relatives out of hospital beds and into ALH facilities [4, 18, 19, 21, 35, 42]. In Cheek and Ballantyne's [19] Australian study of 25 family members, acute care settings were described as hostile environments where older adults were perceived to be occupying “valuable beds.” Hospital providers were described as failing to inform family members of funding limitations that restricted hospital stays until the very end, placing pressure on family members to make life-changing decisions within short timelines [4, 35, 37].

Pressured decisions were found to be particularly difficult for adult children. Edwards et al. [4] found adult children to be under the most stress during the active transition process because of the time-consuming search and selection process. In Cornes et al.'s [42] study, one adult child stated that “it was a couple months in… when the pressure started to build… We went in one day and the Sister came to us and said, ‘are you making any progress with regards to the nursing home?' There was no way I was going to be rushed into making a decision on which nursing home my mother was going to go in… the thing is when you are working full time you have only got the weekends to go and visit these places, and you want to view before you make your decision” (p. 19). While this is an example of a stressed, yet resilient adult child caregiver, her resistance to give in to systemic pressures is rare when compared to other adult children caregivers.

Many are forced to concede to the pressures of moving parents out of hospital in the absence of an alternative housing arrangement by making quick decisions. These decisions may result in poor quality care or from placing older adults into respite care settings only to have them move out shortly thereafter when a more suitable bed becomes available [19, 35]. This finding was supported by Cornes et al. [42] who found that family members experienced anxiety with disrupted living arrangements, payment for interim services, inconvenience with multiple moves, and inadequate or inappropriate care.

### 3.25. Limited Availability of Nursing Homes

Pressured decisions were also common among family members when time came to accept or decline offers for nursing home placement [35]. Others found themselves begging nursing homes with no vacancies to place older relatives in their care [21, 35]. According to Cheek and Ballantyne, [35] family members experienced despair and overwhelming stress and compared dealing with the nursing home system to “fighting a battle.” For example, one study participant stated, “Well, it was a bit like being at war because it was a full-on combat almost. I had to practically batter these places, plead and cry… I do think it was like a war. It was a constant battle and I was utterly fatigued the whole time” (35: p. 229). As a result of limited bed availability, many family members were forced to consider alternatives that were at times considered undesirable, and thus placement became a temporary place to address immediate crises until a more desirable setting became available [21].

### 3.26. Limited Access to Information and a Lack of Support

Family members reported having limited access to information on available resources, support, and the overall process of transitioning older adults into ALH [19, 21, 24–26]. Cheek and Ballantyne [19] described this experience as being “at the mercy of the system,” feeling out of control, being unsure of what to do next and where to turn for help [20]. When coupled with precipitous discharges from hospital, a lack of information led to poor transitions and feelings of discontent among family caregivers [25]. Thus, many identified the need for comprehensive information on LTC options and additional support from healthcare professionals [7, 19, 21, 24, 26, 29, 35].

### 3.27. Influences during the Active Transition Period

Informal and formal support networks play an important role during the active transition process [4, 12, 15, 22, 31]. Positive interactions within support networks assist family caregivers in identifying and weighing pros and cons of transitioning to ALH and gaining access to valuable transition information [31]. Informal validation and recommendation by peers give family members a sense of comfort during the search and selection process and active transition of older adults into ALH [4, 12, 22]. Moreover, close relationships with health and social-care practitioners provide additional support to family members during the decision-making process, making them aware of specialized advanced care services and generating the perception that healthcare practitioners have family members' best interests at heart by performing the role of “carer advocate” to support successful transitions [22, 31]. This finding was particularly evident in Ryan et al.'s [22] study of rural family caregivers who had significant contact with general practitioners, district nurses, and social workers and thus felt a sense of comfort and support that older family members would receive the best possible care from healthcare professionals in their rural communities.

### 3.28. Quality in Nursing Homes

Choosing a nursing home involves a complex process of weighing individual priorities with available services. There are several key priorities for family members when choosing an ALH facility for older adult relatives. Geographic location and proximity to home/family/friends and quality care are consistently identified as the most influential factors affecting the selection process [11, 12, 18, 19, 32, 38, 42, 43]. Quality in nursing homes can be further described to include the kindness of staff, quality of care, level of staff support, nutritious menus, privacy, variability of activity programs, accessibility of services, clean spacious environments, culturally appropriate care, and secured parameters to prevent wandering [11, 12, 18, 19, 38]. Cost was also identified as an important consideration because it had the potential to limit choices [12, 32].

### 3.29. Summary of Section

The active transition period has been described by family members as a time characterized by pressured decisions, feeling rushed, lacking choice and control, having limited communication with health and social service providers, and lack of access to information. Positive interactions with formal and informal support networks can help family members identify pros and cons of transitions and can provide them with access to valuable placement information, advanced care services, and comfort in knowing that family members' best interests are being addressed. Furthermore, family members endure a process of weighing priorities with available services in order to choose appropriate ALH. Geographic location, proximity to family members and friends, and quality care are identified in the literature as the most influential factors associated with transitions.

### 3.30. Posttransition Period

The posttransition period refers to the period of time following the transition of older adults into ALH. It has been described as a period of adjustment, both emotionally and socially, as family members assume new roles and responsibilities while creating a new “status quo” [21].

### 3.31. Emotional Adjustment

Researchers highlight transient relief, guilt, ambivalence, and loneliness as primary emotions experienced by family members during the posttransition period. A general sense of relief is attributed to finding a good home and being grateful for a reasonable housing solution to what is perceived as an insurmountable problem [11, 18, 19, 21, 35, 37]. In fact, family members attribute the finding of a good home to luck rather than effective planning or experience, and this sense of luck is a result of limited control during the process [18, 19, 35].

Feelings of relief about the burden of care being lifted contrasts with self-accusations and feelings of guilt because of family members not performing their duty to care, ambivalence towards the future of their parent, permanence of the transition, and uncertainty regarding whether or not they chose the “right” home [4, 11, 20, 23, 34]. In Ball et al.'s [44] study, one family member illustrated guilt about failing to perform caregiver duties by stating, “I feel really guilty. I promised her I would never put her in a home, that I would let her stay home. I tried, and it just didn't work” (p. 99).

Many expressed guilt towards breaking promises and being unable to help older adults stay at home longer [18, 34]. These feelings of ambivalence resulted in family caregivers continuously evaluating their decisions and in some cases changing their transition decision [4, 7, 29]. Feelings of ambivalence were particularly apparent in Castle's [12] study where 87% of 306 family caregivers indicated that they would do something differently regarding the transition if they were given the opportunity to go back in time, and 77% of these individuals reported that doing things differently would have been extremely useful.

It is important to note that many of these emotions were experienced simultaneously [5, 29]. For example, one family member from Nolan and Dellasega's [29] study reported feeling “sad, because it's the end of life as we know it. Relief, because of the tremendous responsibility. Worry, that the home is the right one and they'll look after him properly” (p. 764). Furthermore, many of these emotions have been documented in the literature as occurring in the pretransition and active transition periods, thus indicating the persistence and complexity of these emotions and the need to support family members as they adjust to this significant life-changing event [5].

### 3.32. Role Adjustment

Transition into ALH impacts roles and responsibilities assumed by family caregivers in the posttransition period [1, 5, 20, 21, 23, 34]. According to Penrod and Dellasega [21], for the majority of caregivers, responsibilities do not cease upon admission; instead, they change form as family members adapt to new systems of care. These responsibilities include visiting the older adult, scheduling extended family members visit on different days of the week to reduce social isolation, furnishing living space to increase familiarity and feelings of belonging, assisting with activities of daily living and instrumental activities of daily living, monitoring nursing care, highlighting individuality and strengths of older adults to staff members, advocating for relatives, and addressing concerns with staff members [11, 20, 23, 34, 38]. Sanderson and Meyers [23] found that this continued commitment to support the needs of older adults stems from a combination of love, guilt, obligation, and responsibility. Further, deeply bonded relationships prior to the transition were perceived to be the foundation for continued support and connection following the transition [20].

### 3.33. Factors Impacting Familial Satisfaction and Adjustment

Familial satisfaction during the posttransition period is contingent upon a number of factors. First, family members feel more at ease and less ambivalent about their decision about place when they observe older adults receiving adequate, quality care [23]. Second, positive interactions with staff members, extended family members, older adults who have been placed in ALH, and other residents also improve familial satisfaction [29, 34, 38]. Third, involvement in nursing home support groups has been found to improve satisfaction by providing a venue for family members to share experiences, learn about resources, and interact with others in similar situations that can empathize with their individual struggles [29, 34].

Dissatisfaction among family members in the posttransition period was associated with negative interactions with family members and friends who questioned their decision to place older adults in ALH. In many cases, these family members and friends were individuals who lacked experience with nursing home placement [34]. Moreover, dissatisfaction was experienced when family members and friends placed a limit on how much they were willing to hear about the caregiver's experience of the transition process [34]. Feelings of dissatisfaction were heightened among family members who experienced a lack of communication or poor interaction with nursing home staff, who at times disregarded the family member's knowledge of and preferences for the older adult and limited their involvement and influence over their care [20].

### 3.34. Summary of Section

The posttransition period has been described as a time of emotional and role adjustment for family members. Families experience a multitude of emotions, most commonly ambivalence, guilt, transient relief, loneliness, and luck. Many continue to provide caregiver support for older adults in ALH. Positive adjustment and satisfaction is achieved through encouraging interactions with ALH staff members, involvement in nursing home support groups, and personal observation of quality care in ALH. Poor adjustment in the posttransition period occurs when family caregivers remain ambivalent regarding their decision about place, lack social support, and experience substandard interactions with ALH staff members.

## 4. Discussion

This scoping review adds to the body of knowledge on family members experiences of transitioning older adult relatives into ALH. It demonstrates that the transition process is complex and has multiple intersecting factors that can result in smooth or difficult transitions into ALH. A majority of family caregivers experience the pretransition and active transition periods as a time of crisis. A lack of planning, information, support, and transparent discussion is at the root of this problem. With more than 2 million Canadian caregivers of older adults, it is essential that services be made available to family members and older adults in order to support gradual transitions that consider all available options for housing and additional support [45]. Options for home care should be made available at subsidized prices to help people remain at home and receive care at home for as long as possible.

This scoping review also illustrates a significant body of literature on the pretransition period from the perspective of family caregivers. There is an apparent need for additional research on the posttransition period, particularly family caregiver adaptation and adjustment socially, economically, and psychologically. Furthermore, there is a need to explore the impact of continued family caregiving in ALH, specifically the various roles (if any) assumed by family members. Interestingly, the articles that reported on the sex of the caregiver participants (*n* = 13) had significantly more representation of female caregivers in the articles reviewed [12, 20, 23, 27, 29, 31–35, 37, 38, 41]. None of the papers adequately reviewed the sex or gender differences in decision-making. Additional research should be done to examine the potential sex and/or gender bias that caregivers' experiences can have on results.

Additional research is necessary for assessing the effectiveness of and models for providing support groups for family members with loved ones in nursing homes. Nolan and Dellasega [29] and Garity [34] have reported positive outcomes associated with family involvement in nursing home support groups and additional research that supports this conclusion may encourage the widespread establishment of support groups to improve familial satisfaction and adjustment in the posttransition period.

Future research should also focus on alternative models of ALH. There are new and innovative housing options available to older adults that differ from traditional nursing and retirement home care models, and these options may offer a means of easing the transition process while maintaining quality of life for older adults and their family members. These models include, for example, community based villages and supportive housing apartments. While these models may be shown to be useful with additional research, it will be important to consider other factors associated with the social determinants of health, particularly cost and out-of-pocket payments which will undoubtedly limit options for older adults of lower income. Nevertheless, additional research on new and innovative models of housing may propel enhancements to existing models, ultimately improving services for older adults.

There is also a need to establish a comprehensive list of existing housing options that can be easily accessed by older adults, family members, and friends. In order to effectively distribute this information, a multimodal approach must be used and should include, for example, providing information in medical offices, community centers, religious institutions, newspapers/community newsletters, and other places of gathering. Strategies for distributing information must be community-specific, user-friendly, and accessible in multiple formats, including electronic and paper copies. Government agencies, such as the Community Care Access Centre, should take a leading role as navigators of the housing system by providing information on ALH, both private and publicly funded, to older adults and their family members in order to facilitate planning, reduce the economic burden of caregiving, and prevent future crisis.

A list of recommendations for choosing ALH and easing the transition process should be developed using the expertise of family caregivers with lived experience of transitioning a loved one into ALH. Weber and Bailey [38] conducted focus groups with 20 family caregivers of older adults with dementia in the United States. Focus group discussion revealed a number of strategies that caregivers could take to ease the transition, such as planning ahead, attending support groups, doing “homework” well in advance of the transition, visiting as many facilities as possible, observing staff members doing their jobs, and communicating with management to determine whether they have an open-door policy. Holding structured focus groups similar to Weber's could help identify a list of recommendations to be shared broadly to help guide family caregivers and older loved ones during the transition process.

Future research should also explore the use of interim care options for older adults as potential ways of trialing specific types of housing and/or providing respite for caregivers. Aubrey-Fletcher [11] and Caldwell et al. [18] recommend convalescing or “holidaying” at nursing homes in order to gain a better understanding of how homes are run, acclimatizing to the facility's culture, and getting to know other residents in order to determine whether a particular home meets the older adult's needs and preferences before committing. This approach also has the potential of helping older adults with cognitive impairments adjust to new environments at a more reasonable pace [18].

## 5. Conclusion

A scoping review of literature published from 2000 to 2014 identified three key time periods involved in the transition to ALH: pretransition, active transition, and posttransition periods. Literature on the pretransition period highlights two extremes of planning: gradual and crisis planning, with the latter representing a majority of family members' experiences during this transition. Several factors precipitate the transition process, including health deterioration of family members and the older adult being cared for and formal/informal influences. The literature highlighted barriers to transition, which include familial conflict, cultural expectations, negative perceptions of long-term care, and suitability of nursing homes.

The active transition period is characterized by pressured and rushed decisions with limited choice, lack of access to information, and communication with providers. This is a time when family members weigh priorities with preferences regarding the transition. Furthermore, positive interactions with formal and informal networks can help family members thrive during the transition period.

The literature describes the posttransition period as a time for emotional and role adjustment. Positive adjustment and satisfaction is maintained through positive interactions with staff members, participation in support groups, and observation of provision of quality care. On the other hand, poor adjustment occurs when family members remain ambivalent, lack social support, lack access to information, and engage in substandard communication with staff members.
